# Professor Patricia Rocco pays tribute to Professor Paolo Pelosi

**DOI:** 10.1186/s13613-023-01151-8

**Published:** 2023-06-24

**Authors:** Patricia R. M. Rocco

**Affiliations:** Laboratory of Pulmonary Investigation, Carlos Chagas Filho Institute of Biophysics, Federal University of Rio de Janeiro, Rio de Janeiro, Brazil


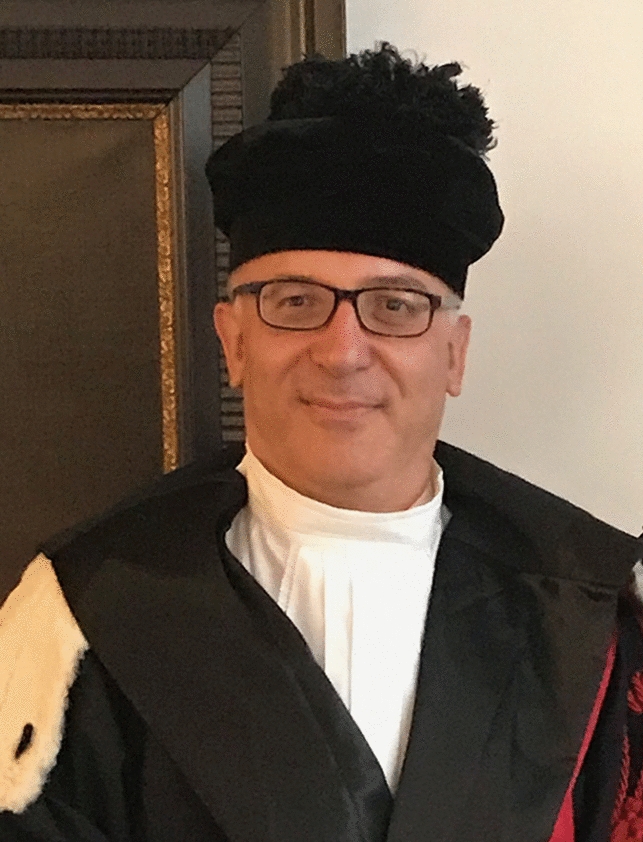


Paolo Pelosi, Professor of Anaesthesia and Intensive Care at the University of Genoa, Italy, died on 30 May 2023 at the age of 60. Paolo was a distinguished scientist, a mentor to countless residents and students, and an exceptional leader and friend.

Paolo grew up in Milan and attended its *Università degli Studi di Milano*, graduating *magna cum laude* in medicine and surgery in 1987. After graduation, he completed his studies first as specialist in anaesthesia and resuscitation in 1991, Clinical Assistant in Anesthesiology at San Gerardo Hospital, Monza, from 1990 to 1993, and then returned to the University of Milan as Research Fellow in Anaesthesiology from 1993 to 1999. He subsequently served as Associate Professor in Anaesthesia and Intensive Care Medicine at the University of Insubria, Varese, from 1999 to 2010; and from 2010 to his death, was Chief Professor of Anaesthesia and Intensive Care and Director of the Specialist School in Anaesthesiology at the University of Genoa, as well as Head of the Anaesthesia and Intensive Care Unit and Director of the Regional Poison Control Centre for Liguria, both at the IRCCS San Martino-IST Hospital in Genoa. He served with distinction as President of the European Society of Anaesthesiology, ESA (2010–2011); first as Secretary and then as Head of the Respiratory Intensive Care Assembly 2 at the European Respiratory Society; and as a board member of the World Federation of Societies of Intensive Care and Critical Care Medicine. Recently, he was elected as Vice President of the Italian Society of Anaesthesia, Analgesia, and Intensive Care (SIAARTI) and as its President for the years 2025–2027, a role he will sadly be unable to take on.

Paolo’s activity has been always evenly divided between bench and bedside. His main research interests were particularly focused on respiratory physiology during anaesthesia and respiratory failure; over the years, he developed several fruitful collaborations with highly reputable research groups around the world. For his work and many achievements, Paolo was awarded the *British Journal of Anaesthesia*/Liverpool Society of Anaesthetists, T. Cecil Gray Medal in 2015 and gave the 2019 Sir Robert Macintosh Lecture at the European Society of Anaesthesiology and Intensive Care. One day before his death, he received the International Career Award in Intensive Care Medicine from the Chilean Society of Critical Care and Emergency Medicine and, in a final act of dedication to the field, gave a lecture—albeit virtually—at the Society’s opening session. Other honours and awards, too numerous to list here, included honorary membership of several scientific societies in the fields of anaesthesiology and intensive care; corresponding membership of the National Academy of Medicine in Brazil; and Fellowship of the European Respiratory Society.

Paolo and I first met at the European Respiratory Society Congress 20 years ago, where we discussed several projects associated with mechanical ventilation strategies for acute respiratory distress syndrome. We have worked together ever since, publishing more than 200 papers and book chapters. We met every year in Brazil and Italy, organized several meetings, and travelled across Brazil and other countries. His passion for medicine, generosity of spirit, and extraordinary intellect will be missed.

Paolo will be remembered not only as an outstanding scientist and physician, but also as someone who always gave his time and expertise generously, no matter how busy, to listen, to advise, and to help—always sociable, always with a smile, and always letting his terrific sense of humour shine through. His huge legacy includes advances in mechanical ventilation; the mentorship and training of innumerable clinical and non-clinical trainees and fellows; and many grateful patients over his decades of practice. The legion of physicians, residents, students, and researchers associated with Prof. Pelosi, while deeply feeling the vacuum created by his demise, shall do their best to carry this legacy forward.

He will be greatly missed by his friends, colleagues, mentees and, of course, by his beloved family: his wife Roberta, his son Matteo, and his lovely mother Elisabetta.

Rest in peace, my dear friend Paolo.

